# Early establishment of trees at the alpine treeline: idiosyncratic species responses to temperature-moisture interactions

**DOI:** 10.1093/aobpla/plw053

**Published:** 2016-08-17

**Authors:** Hannah Loranger, Gerhard Zotz, Maaike Y. Bader

**Affiliations:** 1 Functional Ecology of Plants, Institute of Biology and Environmental Sciences, University of Oldenburg, D-26111 Oldenburg, Germany; 2 Ecological Plant Geography, Faculty of Geography, University of Marburg, Deutschhausstraße 10, D-35032 Marburg, Germany

**Keywords:** Alpine treelines, climate change, early seedling survival, germination, temperature–moisture interactions, time-to-event analysis

## Abstract

On a global scale, temperature is the main determinant of arctic and alpine treeline position. However on a local scale, treeline form and position vary considerably due to other climatic factors, tree species ecology and life-stage-dependent responses. For treelines to advance poleward or uphill, the first steps are germination and seedling establishment. These earliest life stages may be major bottlenecks for treeline tree populations and will depend differently on climatic conditions than adult trees. We investigated the effect of soil temperature and moisture on germination and early seedling survival in a field experiment in the French Alps near the local treeline (2100 m a.s.l.) using passive temperature manipulations and two watering regimes. Five European treeline tree species were studied: *Larix decidua*, *Picea abies*, *Pinus cembra*, *Pinus uncinata* and *Sorbus aucuparia*. In addition, we monitored the germination response of three of these species to low temperatures under controlled conditions in growth chambers. The early establishment of these trees at the alpine treeline was limited either by temperature or by moisture, the sensitivity to one factor often depending on the intensity of the other. The results showed that the relative importance of the two factors and the direction of the effects are highly species-specific, while both factors tend to have consistent effects on both germination and early seedling survival within each species. We show that temperature and water availability are both important contributors to establishment patterns of treeline trees and hence to species-specific forms and positions of alpine treelines. The observed idiosyncratic species responses highlight the need for studies including several species and life-stages to create predictive power concerning future treeline dynamics.

## Abstract (precis)

Alpine treelines globally may move upslope due to climatic warming. Such movement would need, as the first steps, seed germination and seedling establishment above current treelines. These processes were studied experimentally in five common European treeline tree species. Surprisingly, each species responded very differently to moisture and temperature gradients, with positive and negative responses possible. These results match the heterogeneity observed in treeline dynamics and spatial patterns globally. They strongly emphasize the need for species-specific parameterisations in predictive models of treeline responses to climatic change.

## Introduction

Treelines are conspicuous transition zones between two very different vegetation types. There is a growing concern about how global climate change may affect these systems, and as a consequence much attention has been drawn to both alpine and arctic treeline ecotones in recent years. Treelines could represent a distinct indicator of climate warming since temperature is recognized as the main determinant of treeline position on a global scale, roughly following a common isotherm of 5–7 °C mean growing season temperature (Körner and Paulsen 2004). Many studies show recent advances of treelines poleward and to higher elevations, as well as increasing radial growth of the trees forming these ecotones (Rolland et al. 1998; Kullman 2007; Shiyatov et al. 2007; Qi et al. 2015). However, stable or receding treelines have been found (Harsch et al. 2009), and treeline position may vary considerably at a local scale (Holtmeier and Broll 2005; Case and Duncan 2014). Such local variations can be due to locally varying environmental conditions unrelated to temperature such as precipitation (Holtmeier and Broll 2005), tree species ecology (Körner and Paulsen 2004; Trant and Hermanutz 2014) and life-stage dependent environmental dependencies (Barbeito et al. 2012; Greenwood et al. 2015).

These abiotic and biotic factors may also interact with temperature to determine the form and dynamics of a treeline at a specific site. For example, the consequences of moisture deficits—which can be positively linked to climate warming—have been shown to override positive temperature responses with respect to growth (Barber et al. 2000; González de Andrés et al. 2015) and regeneration (Barton 1993; Daniels and Veblen 2004; Moyes et al. 2013). In such cases, treeline shifts may depend more on the interactions of temperature and water availability than on their absolute values (Ohse et al. 2012) and may differ between landscape positions accordingly (Elliott and Cowell 2015). It is commonly observed that tree cover is slow or unable to expand to its ultimate thermal boundary (Holtmeier 2009). The underlying mechanisms remain however difficult to disentangle and there is an urgent need for quantitative assessments of the specific environmental conditions and associated mechanisms preventing the establishment of different tree species beyond current treelines.

Treelines represent distributional boundaries for an entire life-form—the tree. Consequently, ecosystems above the treeline differ fundamentally from those below, e.g. in regard to soils and microclimates (Sullivan and Sveinbjörnsson 2010; Thébault et al. 2014). This presents particular challenges for a successful tree regeneration and establishment in the treeline ecotone and beyond, as required for an upward distributional shift. Previous studies have shown that traits essential for regeneration such as the number of seed-bearing fruits or the number of viable seeds often decrease with increasing elevation, thereby reducing the probability of seedling establishment especially above treeline (Cuevas 2000; Kroiss and HilleRisLambers 2015). Although seed production and dispersal are critical prerequisites for tree regeneration, subsequent germination and seedling establishment have also been widely recognized as potential life-history bottleneck of treeline tree populations (Stevens and Fox 1991; Germino et al. 2002; Smith et al. 2003; Johnson et al. 2011).

Germination represents the earliest, critical life-stage transition and should thus be subject to strong natural selection (Baskin and Baskin 2001). Furthermore, the conditions during germination also influence the phenotypic expression of post-germination traits, thereby affecting later seedling performance (Donohue et al. 2010). Once successfully germinated, the germinant enters the most vulnerable life-stage of a tree, characterized by the highest mortality of the whole life-cycle (Cui and Smith 1991; Johnson et al. 2011). Most studies investigating environmental dependencies of both early life-stages find that favourable conditions are concordant (i.e. the same conditions are favourable for both seed and seedling), though others report conflicting requirements (reviewed in Schupp 1995). Hence, it remains unclear to what extent the effects of environmental conditions on regeneration success are life-stage specific.

The natural seedling distribution in treeline ecotones, a result of limitations to both early life-stages, is often found to be related to stress-reducing site features such as reduced sky exposure or shelter from strong winds (Germino and Smith 1999; Smith et al. 2003; Batllori and Camarero 2009). Furthermore, seedling density often decreases with elevation (Cuevas 2000). Both observations are in line with the view that the lack of safe sites (sensu Harper 1977) and the harsh climatic conditions in the alpine zone might restrict the regeneration of treeline trees (Tranquillini 1979). Most research focusing on the earliest stages of tree regeneration at treeline sites used germinating seeds principally to study subsequent young seedling survival and physiology (Germino and Smith 1999; Germino et al. 2002; Moyes et al. 2013), or lumped germination and subsequent seedling survival due to long observation intervals (Zurbriggen et al. 2013). Others explicitly including germination responses at finer temporal scales mainly used elevation gradients to study recruitment responses, without actively manipulating microclimate (Ferrar et al. 1988; Castanha et al. 2012). As the process of germination differs in genetic regulation and environmental sensitivity from survival mechanisms in emerged seedlings and may thus be evolutionarily decoupled, it is important consider these two life stages separately. For that, seedling emergence and subsequent survival need to be monitored frequently following individual seeds (and seedlings). Moreover, potentially complex interactions of microclimatic variables and responses of early tree establishment require experimental manipulations of more than one limiting factor. To our knowledge, no study has ever addressed germination and subsequent survival separately while manipulating multiple environmental factors in a field experiment on regeneration limitations at treelines.

To summarize, any attempt to understand current treeline patterns and positions mechanistically and to predict their future dynamics requires investigating local treelines with regard to microclimate-, species- and life-stage-specific responses. In this study, within a single field experiment, we assessed the germination and early seedling-establishment responses of five important European treeline tree species to the variation of two important microclimatic variables, temperature and moisture. Accordingly, we asked the following research questions: (i) Do responses of treeline trees to microclimatic conditions vary with life stage, i.e. during germination and early seedling establishment? (ii) Do temperature and water availability interact to determine germination and early seedling survival? (iii) Do different treeline tree species show consistent responses to temperature and moisture conditions? In addition to the field experiment, we monitored the germination response to low temperatures under controlled conditions in growth chambers for three of the five study species. This allowed us to assess temperature responses along a defined gradient and at a finer temporal scale, complementing the results from the more complex field study.

## Methods

### Effect of soil moisture and temperature under field conditions

***Study site and species*.** A common garden germination experiment was set up in the experimental garden of the alpine research station Joseph Fourier in the French Alps near the local treeline (Lautaret Pass, 2100 m a.s.l., 45°02′N, 6°24′E). The site is situated in a climatic transition zone between the wet outer Alps and the dry inner Alps (Ozenda 1988), with 11 °C as the mean temperature of the warmest month (July) and an average annual precipitation of 1230 mm (Choler et al. 2001). The study species comprise four important treeline-forming conifers of the European Alps: *Larix decidua*, *Picea abies*, *Pinus cembra*, *Pinus uncinata* as well as the deciduous angiosperm *Sorbus aucuparia*, which also occurs up to treeline elevation (Brändli 1998). Seeds of subalpine origin from the inner Alps were obtained from a commercial seed producer (Herzog Baum, Samen und Pflanzen GmbH, Gmunden, Austria) and a forestry office (Kantonaler Forstgarten Rodels, Rodels, Switzerland), except for seeds of *S. aucuparia*, which were available only from montane origin in Hungary (Table 1). Information on seed germinability—either provided by the supplier or determined from standard germination trials—was used to adjust the seed quantity sown per plot (Table 1). Relatively large seed quantities were sown to account for a potentially lower germination success under field conditions, allowing a reliable estimation of germination proportions and ensuring a sufficient number of seedlings to monitor subsequent survival. Due to time constraints, the seed quantity had to be reduced in the third experimental block.

**Table 1.** **** Seed characteristics and seed quantities used in the germination field experiment

Species	Elevation of seed source (m a.s.l.)	Germinability (%)	Seed quantity (no.)
*L. decidua*	1800–2000	33 %^a^	240 (120)
*P. abies*	1100–1400	63 %^b^	120 (60)
*P. cembra*	1300-2850	89 %^b^	30
*P. uncinata*	2100	78 %^a^	120 (60)
*S. aucuparia*	400–1400	86 %^b^	120 (60)

^a^Germinability of seed lot determined by own standard germination trial (winter 2012–13).

^b^Germinability of seed lot provided by seed supplier.

Seeds originated always from the inner Alps, except for *S. aucuparia*, which was only available from Hungary. Numbers in brackets indicate reduced seed quantity sown in the third experimental block. For *P. cembra* seed quantity was always limited to 30 seeds per row due to the large seed size of 1–1.5 cm.

***Experimental design.*** Fifteen experimental plots (70 × 30 cm) were arranged in three blocks to account for spatial heterogeneity, with ∼5 m distance between the centres of two adjacent blocks and 20 cm distance between plot edges. All blocks were enclosed by a 60 cm high wire-mesh fence as protection against rodents. The vegetation cover on the plot surface was removed and plots were excavated to a depth of 15 cm to remove rocks and large roots from the soil. The soil of plots from the same block was then mixed and returned to the plots. This procedure was done both to create a homogenous growth substrate within blocks and to remove the effects of biotic interactions such as competition or facilitation by neighboring vegetation, allowing us to focus on abiotic factors. In October 2013, seeds were sown in one row of 60 cm length per species, allowing 3 cm spacing between rows and 5 cm plot margin. Rows were randomly assigned to one of the five species. Seeds were sown in 2-cm deep grooves, distributing seeds evenly with the fingertips and closing up the soil. Seeds of *P. cembra* were limited to 30 seeds per row and placed individually due to their large size.

In spring of 2014, two watering regimes and two types of installations for passive temperature manipulation, open-top chambers (OTCs; passive warming) and shade roofs (passive cooling), were set up to create a gradient of soil temperature and soil moisture across all experimental plots. OTCs were conceived as hexagons (Marion et al. 1997; *r* = 80 cm, *h* = 30 cm) with 3-mm thick acrylic glass panels transmitting 92 % of solar radiation, including UV. Shade roofs consisted of a plot-sized wooden frame covered with a shade net, providing 70 % shade on the plot surface but allowing rain water to pass. The roofs were supported by four 30 cm high metal poles at the plot corners with 20 cm shade net curtains on each side to prevent the penetration of low-angle sunshine. Control and warming treatments were crossed with a watering treatment, with watered plots receiving 3 mm irrigation on days without rainfall throughout the study period (in total adding up to 35 % of the May–September precipitation in 2014). Since cooling through shading was already expected to decrease evapotranspiration and thus increase soil moisture, this treatment was not included in the additional-watering regime. All five microclimate treatments (control (C), watered control (C + W), passive warming (OTC), watered passive warming (OTC + W), passive cooling (Sh)) were replicated in each of the three rexperimental blocks. Treatments were initiated directly after snowmelt in a block-wise manner due to a highly heterogeneous snow cover, with about 4 weeks between the start in the first block (mid-April) and the last block (mid-May). The study period covered the complete growing season of 2014 from snowmelt to early September.

***Microclimate.*** The soil moisture content (soil MC) (%) was measured monthly with a hand-held sensor inserted 15 cm in each plot centre (TRIME-PICO64, IMKO Micromodultechnik, Ettlingen, Germany), while the soil temperature was measured at 5 cm depth with external sensors of permanently installed temperature loggers (Hobo ProV2, Onset Corp, Bourne, MA, USA). Since comparative measurements in the same microclimatic treatments in 2013 had shown that there is no significant temperature difference between watered plots and their control (C vs. W: *P* = 0.43; OTC vs. OTC + W: *P* = 0.76; 2-sample *t*-test, *n* = 5), soil temperature was only recorded in the three temperature-relevant treatments C(+W), OTC(+W) and Sh. In each block, a temperature logger was assigned by chance to either a watered plot or its control and then inserted in the plot centre, recording data in 30 min intervals.

Integrated variables were calculated to obtain a quantitative gradient of both soil moisture and soil temperature across all plots. Mean soil MC (%) was calculated as the seasonal average of 4 monthly measurements for all plots, giving a soil MC gradient with 15 observation points. Soil heat accumulation relevant for germination and seedling survival was expressed in growing degree days with a base temperature (*T*_b_) of 2 °C, which is the lower temperature limit for germination of at least two of the study species (Løken 1959; Barclay and Crawford 1984). In the plots where temperature was recorded, the number of soil growing degree days (soil GDD; no.) was calculated by summing up the positive differences between temperature recordings and the base temperature over the whole study period and dividing the results by the measurement interval fraction of a day (30 min/24 h = 48, Equation 1), giving a soil GDD gradient with nine observation points.

(1) $$\text{GDD}=\frac{\sum_{i/T_{\text{i}}>T_{\text{b}}}\left(T_{\text{i}}-T_{\text{b}}\right)}{48}$$

for temperature recordings (*T*_i_) higher than the base temperature (*T*_b_).

***Seedling survey.*** Emerging seedlings were recorded weekly and each marked with a coloured pin to allow the assessment of individual survival. Since hypogeous germination could not be monitored directly, seedling emergence was used as a proxy for total germination success by calculating the proportion of sown seeds that emerged as seedlings (including subsequently dead individuals). Seedling survival was calculated as the proportion of emerged seedlings that survived until the end of the growing season.

### Effect of temperature under controlled conditions

Complementing the field experiment, a standard germination trial investigating the effect of low temperatures on germination was performed for three of the five studied species under controlled conditions in growth chambers (Economic Delux Snijders Scientific, Thermotec, Weilburg, Germany) in winter 2014–15. Batches of 25 seeds of *P. uncinata* and *P. abies* and 60 of *L. decidua* were placed on moist paper tissue in sealable plastic boxes (volume = 280 mL) with six replicates for every temperature treatment. Four low-temperature treatments comprised constant regimes of 16, 12, 8 and 4 °C and all treatments included a 12-/12-h light–dark-cycle. As control treatment we used the settings 20/15 °C 12-/12-h, previously identified as optimal for the same seed lots (germination percentages: *P. uncinata*: 79 %, *P. abies*: 63 %, *L. decidua*: 33 %). Germination boxes were rotated on their tray every other day to assure homogeneous temperature exposure.

Successful germination was recorded for every single seed in 2- to 3-day intervals as 1-cm growth of the radicle. Germinated seedlings were removed. The temperature treatments were discontinued when a species showed no further germination for 2 weeks. The remaining seeds of the two warmest treatments (control, 16 °C) were all non-viable, their soft texture and liquid discharge indicating decay of the embryo, unambiguously indicating maximum germination. Seeds in the three cooler treatments (12, 8 and 4 °C) still showed a very slow increase in germination after 11 weeks so that remaining, healthy-looking seeds were transferred to the control temperature for another 3 weeks to test their viability.

### Statistical analysis

All analyses were performed using R 3.2.1 (R Core Team 2015). Germination and survival in the field experiment at the end of the growing season 2014 were expressed as a 2-column vector of counts of successes and failures per species per plots and analysed with binomial generalized linear models (GLM), including block, soil MC and soil GDD and the interaction soil MC: soil GDD as explanatory variables. Using these continuous gradients as explanatory variables instead of the treatments allowed differentiating the relative effects of soil temperature and moisture as well as detecting potential interactions between them. In cases of overdispersion, the standard errors were corrected by using a quasi-GLM model (Zuur et al. 2009). Non-significant terms were removed from the full models in a backwards stepwise approach. To facilitate interpretation, significant interactions between soil MC and GDD are shown graphically by plotting the predicted values from the model along the whole range of one of these two variables (on the x-axis) and for three fixed values of the other variable: low (25 % quartile), intermediate (median) and high (75 % quartile). The variable chosen to represent the x-axis was for each final model the one with the lower *P*-value. Note that the resulting curves are predictions from the models so that they do not directly relate to specific data points; sections of variables which were not measured are extrapolations of the models. Since temperature and moisture extremes were also linked to reduced light intensities by the shading roofs, we evaluated the potentially confounding effect of light by performing an additional analysis excluding the three shaded plots.

Germination data of the growth chamber experiment were analysed using time-to-event analysis (McNair et al. 2012) by first assessing the random variation among replicates with a Cox proportional-hazards model including a frailty term. There was no evidence of variability in frailty levels for any of the three species in any temperature treatment so that data from the six replicates could be pooled. Non-parametric time-to-event analysis was then used to compare temperature treatment differences in the germination pattern of each species with a log-rank test using the survdiff—function in R (survival library; Therneau 2015). Results give test statistics and significance levels of group (temperature treatment) pairwise comparisons for each species. The *P*-values were Holm-adjusted to account for family-wise error rates in multiple comparisons. Treatment differences of survivor functions were graphically displayed by showing the inverse Life-table estimates of survivor functions with point-wise 95 % confidence intervals computed with the R-function lifetab (KMsurv library; Klein and Moeschberger 2012).

## Results

### Field experiment: soil moisture and temperature effects on early establishment

**Figure 1.**
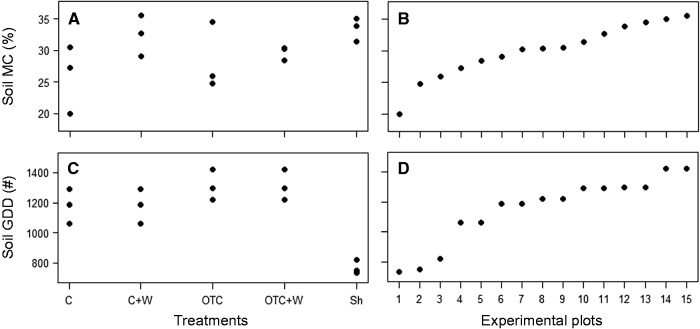
Microclimatic conditions in the field experiment with manipulations of soil moisture (watering, shading) and soil temperature (passive warming and cooling treatments). Shown are the integrated microclimate variables mean soil moisture content (soil MC; %) and total number of soil growing degree days >2 °C (soil GDD; no.) per treatment (A, C; *n* = 3) and the respective gradient of soil moisture and soil temperature over all 15 experimental plots (B, D; sorted by y-axis value, so order differs for soil MC and soil GDD). Treatment abbreviations indicate: C, Control; W,  watered control; OTC, passive warming (open top chamber); OTC + W, watered passive warming; Sh, passive cooling (shading roof).

***Soil microclimate.*** The soil MC gradient ranged from 20 to 35.5 %, with the cooling treatment being the wettest (mean = 33.4 % ± 1.9 SD, *n* = 3) and the non-watered control the driest (mean = 25.8 % ± 5.3 SD, *n* = 3) (Fig. 1), with consistent relative differences between treatments. As expected, heat accumulation of the soil was highest in the warming treatment (mean = 1313 GDD ± 101 SD, *n* = 3) and lowest in the cooling treatment (mean = 769 GDD ± 46 SD, *n* = 3). The complete gradient over the nine measured plots ranged from 733 to 1421 GDD (Fig. 1).

***Seedling emergence and survival.*** Maximum seedling emergence (%) at the end of the growing season under field conditions was invariably lower than germination under optimum conditions in a standard germination trial (*L. decidua*: 25 %, *P. abies*: 40 %, *P. cembra*: 63 %, *P. uncinata*: 59 %, *S. aucuparia*: 23 %; **see Supporting Information—Fig. S1**). Overall, seedling survival at the end of the growing season exceeded an average of 50% for all species, but differed considerably among species (*L. decidua*: 53 %, *P. abies*: 65 %, *P. cembra*: 94 %, *P. uncinata*: 72 %, *S. aucuparia*: 67 %; **see Supporting Information—Fig. S1**).

**Figure 2.**
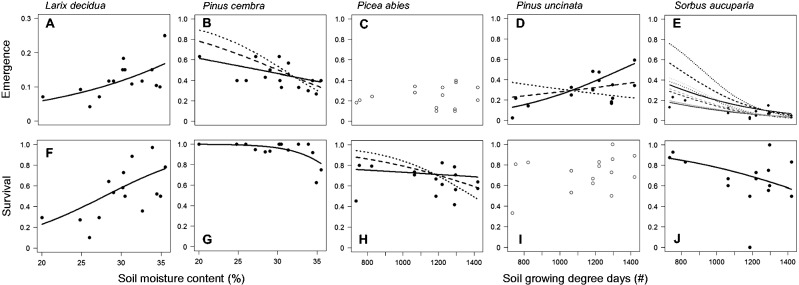
Seedling emergence as proportion of sown seeds (A–E) and survival as proportion of emerged seedlings (F–J) for the five study species in response to soil moisture (soil MC; %), soil temperature (soil GDD, no.) or the interaction of both. Shown are binomial GLM for significant responses, non-significant responses are displayed as open circles for observed values. Significant interactions are shown using fixed values of soil GDD (soil MC) plotted along the complete range of soil MC (soil GDD) for *P. cembra* (*P. uncinata*, *P. abies, S. aucuparia*), line types indicating: dotted, low intensity; dashed, intermediate intensity; solid, high intensity. Note that the resulting curves are predictions from the models so that they do not directly relate to specific data points. The significant block effect in seedling emergence of *S. aucuparia* is displayed by varying hues of grey: black, early snowmelt date (block 1); medium grey, intermediate snowmelt date (block 2); light grey, late snowmelt date (block 3).

The responses of seedling emergence and first-season survival in the five study species to soil moisture and soil temperature were highly idiosyncratic. Although higher soil moisture had a positive effect on both stages of early establishment in *L. decidua*, it had a generally negative effect on *P. cembra* (Fig. 2, Table 2). Similarly, higher soil temperature generally positively affected seedling emergence in *P. uncinata* while having a negative effect on both stages of early establishment in *S. aucuparia* (Fig. 2, Table 2). Within species, however, there was consistency in the climate variable that had the strongest effect and in the direction of this effect between seedling emergence and survival. There were significant interactions between soil temperature and moisture in (i) the emergence of *P. cembra*, *P. uncinata* and *S. aucuparia*, as well as (ii) the survival of *P. abies* (Fig. 2, Table 2): the negative effect of high soil moisture was reduced (Fig. 2B and E) or even inversed (Fig. 2D and 2H) as temperature increased. Conversely, the negative effects of high temperature were reduced (Fig. 2B and E) or reversed (Fig. 2D and H) as the soil MC increased. Finally, a block effect in seedling emergence of *S. aucuparia* indicated that emergence was significantly higher in the block with earlier snowmelt (Fig. 2E).

**Table 2.** **** Summary of binomial GLM testing the effect of soil microclimate (soil MC, soil GDD) on the proportions of germination and subsequent survival at the end of the growing season 2014 of five treeline tree species grown in a field experiment in the french alps at 2100 m

	Block	Soil MC	Soil GDD	Soil MC: Soil GDD
***L. decidua***				
Germination	(*F*_2,9_ < 0.01)^1^	***F*_1,13_ = 8.18** * **↑**	(*F*_1,12_ = 0.31)^3^	(*F*_1,11_ = 0.25)^2^
Survival	(*F*_2,9_ = 0.96)^1^	***F*_1,13_ = 7.25** * **↑**	(*F*_1,12_ = 1.28)^3^	(*F*_1,11_ = 0.27)^2^
***P. cembra***				
Germination	(*χ*^2^_2_ = 4.49)^1^	***χ*^2^_1_ = 5.89** * **↓**	***χ*^2^_1_ < 0.01**	***χ*^2^_1_ = 4.91** * **↑**
Survival	(*χ*^2^_2_ = 3.01)^2^	***χ*^2^_1_ = 10.83** *** **↓**	(*χ*^2^_1_ = 1.14)^3^	(*χ*^2^_1_ = 0.52)^1^
***S. aucuparia***				
Germination	***χ*^2^_2_ = 8.1** *	***χ*^2^_1_ = 1.32**	***χ*^2^_1_ = 36.02** *** **↓**	***χ*^2^_1_ = 6.99** ** **↑**
Survival	(*χ*^2^_2_ = 1.38)^1^	(*χ*^2^_1_ = 0.06)^3^	***χ*^2^_1_ = 8.85** ** **↓**	(*χ*^2^_1_ = 0.36)^2^
***P. uncinata***				
Germination	(*F*_2,9_ = 0.1)^1^	***F*_1,11_ = 6.84** * **↑**	***F*_1,11_ = 21.62** *** **↑**	***F*_1,11_ = 5.11** * **↑**
Survival	(*F*_2,10_ = 0.5)^2^	(*F*_1,13_ = 1.86)^4^	(*F*_1,1 2_ = 2.46)^3^	(*F*_1,9_ =0.08)^1^
***P. abies***				
Germination	(*F*_2,12_ = 1)^4^	(*F*_1,10_ = 0.53)^2^	(*F*_1,11_ = 1.03)^3^	(*F*_1,9_ = 0.08)^1^
Survival	(*χ*^2^_2_ = 0.85)^1^	***χ*^2^_1_ = 0.16**	***χ*^2^_1_ = 0.55**	***χ*^2^_1_ = 5.75** * **↑**

Rows give complete models with *χ*^2^- or *F*-values (for GLM and quasi-GLM, respectively) for germination and survival data at the end of the growing season 2014 with the explanatory variables block, soil MC, soil GDD >2 °C and their interaction (Soil MC: Soil GDD) in the order tested in the model; non-significant variables (given in parenthesis) were removed from the models based on G- or F-tests, respectively, in a stepwise process with superscripts indicating the order in which they were removed. The minimum adequate model is given in bold and significance levels are indicated as: *P* < 0.1,

* *P* <  0.05,

** *P* < 0.01,

*** *P* <  0.001. Arrows indicate whether partial slopes are positive or negative.

An additional analysis excluding the shaded plots showed that effects found when including all plots were generally maintained even on this shortened temperature and moisture gradient, with one exception: the significant negative temperature effects on emergence and survival in *S. aucuparia* disappeared **[see Supporting Information—Table S2]**.

### Growth chamber experiment: germination response to low temperatures

**Figure 3.**
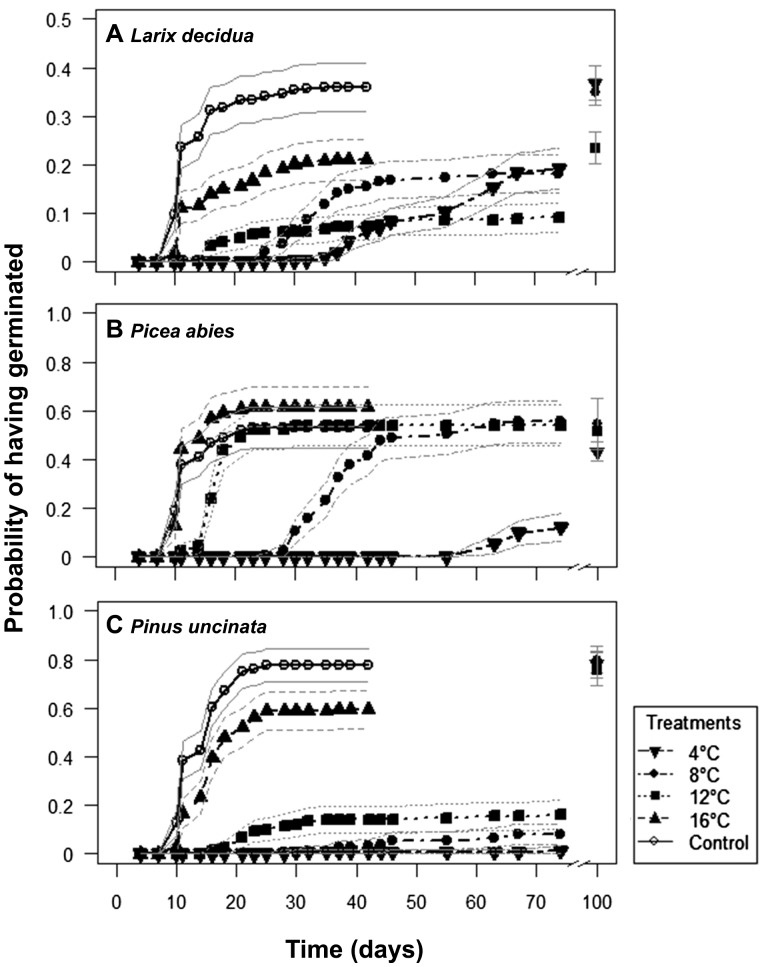
Inverse Life-table estimates of the survivor functions of germination data from a growth chamber experiment representing the probability of having germinated at four low temperature treatments and a control (22/15 °C, 12/12 h) over time, including seeds of *L. decidua* (A), *P. abies* (B) and *P. uncinata* (C). Seeds were pooled over replicates (*n* = 6) giving a total of 360 (*L. decidua*) or 150 (*P. abies*, *P. uncinata*) seeds, respectively. Grey lines represent point-wise 95 % confidence intervals for each treatment. Maximum germination in the control and 16 °C-treatments was achieved after 42 days, with all remaining seeds being non-viable. After 11 weeks, remaining healthy-looking seeds in the three lower temperature treatments (12, 8 and 4 °C) were transferred to control conditions to test their viability. Respective treatment symbols at day 100 give mean ± SD of final germination success after three additional weeks of control conditions.

Germination of the three species was significantly reduced by decreasing temperatures, but with species-specific differences. In *L. decidua* the results of the survivor functions differed significantly mainly between the three highest temperature treatments, showing a 10 % decrease of germination probability per treatment (Fig. 3A, Table 3). In contrast, in *P. abies*, the results of the survivor functions principally differed at the lower end of the temperature gradient (Fig. 3B, Table 3), where differences mainly arose from an increasing delay in the onset of germination. Only for the 4 °C treatment the probability of having germinated was significantly lower (∼30 % lower) at the end of the experiment than in all other treatments, though its slope was still positive, potentially indicating a further increase a longer time span (Fig. 3B). In *P. uncinata*, the most important decrease (∼30 %) in the probability of having germinated occurred at intermediate temperatures, as was shown by highly significantly different results of survivor functions between the 12 and 16 °C treatments, while at the high and low end of the gradient, results were statistically indistinguishable (Fig. 3C, Table 3).

**Table 3.** Summary of log-rank tests comparing the survivor functions of seeds of three treeline tree species germinated under controlled conditions in growth chambers in four permanently low temperature treatments and a control (15/22 °C 12/12 h).

Treatment comparisons	*L. decidua*		*P. abies*		*P. uncinata*	
	*χ*^2^_1_	*P*	*χ*^2^_1_	*P*	*χ*^2^_1_	*P*
4 vs. 8 °C	0.1	1	**61.9**	<0.001***	7.4	0.17
4 vs. 12 °C	*15.5*	*0.08* **.**	**71.8**	<0.001***	**20.8**	0.01*
4 vs. 16 °C	0.8	1	**89.2**	<0.001***	**125.0**	<0.001***
4 °C vs. Control	**32.1**	<0.01**	**64.3**	<0.001***	**195.0**	<0.001***
8 vs. 12 °C	11.4	0.15	11.3	0.15	5.2	0.3
8 vs. 16 °C	0.14	1	**28.9**	<0.01**	**100.0**	<0.001***
8 °C vs. Control	**38.8**	<0.001***	11.2	0.15	**177.0**	<0.001***
12 vs. 16 °C	**21.0**	<0.001***	14.0	0.16	**71.7**	<0.001***
12 °C vs. Control	**80.5**	<0.001***	3.6	0.42	**147.0**	<0.001***
16 °C vs. Control	**22.4**	**0.01** *	1.1	1	*17.3*	*0.07*

Data were pooled over replicates giving a total of 360 (150) seeds for *L. decidua* (*P. abies*, *P. uncinata*), respectively. Rows show results of group (temperature treatment) pairwise comparisons for each tested species presenting *χ*^2^-values and Holm-adjusted *P*-values; significant differences are given in bold, marginally significant differences are given in italics; significance levels are indicated as: *P* < 0.1,

* *P* < 0.05,

** *P* < 0.01,

*** *P* < 0.001.

Viability tests for seeds that did not germinate after 11 weeks in the lower temperature treatments (12, 8 and 4 °C) showed that seed viability was generally not reduced. In almost all cases, a similar germination success as in the control treatment was achieved after three additional weeks under control conditions (Fig. 3). Only the seeds of *L. decidua* coming from the 12 °C treatment showed a substantial (∼10%) reduction in their germination success (Fig. 3A).

## Discussion

Our results show that the early establishment of the focal treeline tree species is affected by temperature and water availability in a very idiosyncratic manner. However, the importance of both climate factors and the direction of their effect on germination and survival tended to be consistent over both stages of early establishment within each species. Interactions of both climate variables indicated that the sensitivity to one factor often depends on the intensity of the other.

### Consistency of limiting factors during early establishment

The consistent effect of microclimate over the life-stage transition from germination to first-year seedling survival (Fig. 2) is in accordance with previous studies (Ferrar et al. 1988; Castanha et al. 2012). This is of particular importance since range limits are primarily imposed during these most critical life-stages (Grubb 1977; Harper 1977). In this context, a high level of consistency over two critical early life-stages will reduce regeneration restrictions arising from seed-seedling conflicts. On the other hand it should increase the impact of relatively stable limiting environmental factors, which could be particularly restricting for regeneration in the harsh conditions of a species’ distributional range edge. In contrast, a variable factor such as irregular freezing events during the growing season can be temporarily decoupled from a short, susceptible life-stage such as germination, but is more likely to affect the longer subsequent stage of the young seedling (Shen et al. 2014). Hence, the degree of concordance or conflict in the environmental requirements between seed and seedling can have a direct impact on the quantity and the distribution of recruits (Schupp 1995).

Yet for two species, *P. abies* and *P. uncinata*, only one of the two studied life-stages showed a significant response (Fig. 2). This might indicate a change in their susceptibility to the two microclimatic factors during early establishment, which is supported by a capability of germinating under a large range of conditions for *P. abies* (Løken 1959 and Fig. 3B) and a relatively resistant seedling stage in *P. uncinata* (Batllori et al. 2010).

### Temperature, moisture and their interactions driving early establishment success

In our growth chamber experiment decreasing temperatures invariably reduced germination (Fig. 3), but germination success of *L. decidua* and *P. abies* was still considerable at low temperatures (Fig. 3A and B). This agrees well with the results of our field experiment, where temperature did not affect seedling emergence of *L. decidua* and *P. abies*. Conversely, *P. uncinata* was particularly temperature-sensitive in the growth chambers and showed a positive temperature response of seedling emergence in the field, confirming the consistency between both experiments.

Our field experiment further revealed that almost all seedling emergence and survival responses were sensitive to water availability, though these responses often showed an interaction with temperature. In *L. decidua* moisture was even the only significant variable, implying that depending on the species, temperature may play rather a subordinate role in limiting early establishment. Our findings thus add to a growing body of evidence that other factors than temperature alone, e.g. water availability, determine seedling distributions at alpine treelines (Ferrar et al. 1988; Sullivan and Sveinbjörnsson 2010; Greenwood et al. 2015; Kroiss and HilleRisLambers 2015; Moyes et al. 2015). Although germination often requires relatively high moisture conditions as environmental cue and to set the necessary physiological processes in motion (Baskin and Baskin 2001), young seedlings depend on it for a longer period of time due to their generally shallow, simple rooting system and their large, transpiring surface area relative to the low water storing capacity (Johnson et al. 2011). Both early life-stages are thus much more affected by water shortage than established trees, potentially creating a bottleneck for regeneration in treeline ecotones, where moisture accumulation and water holding capacities can be spatially highly variable (Holtmeier and Broll 2005).

An important result of this study is that the effects of both temperature and moisture availability on early establishment cannot be decoupled from one another. Especially the combination of opposite extremes, e.g. high temperatures at low soil moisture or low temperatures at high soil moisture had limiting effects on both early life-stages of the study species. Both combinations have previously been shown to restrict tree development, either by temperature-induced moisture stress (Barber et al. 2000; Lloyd and Bunn 2007) or cold soil conditions and insufficient aeration limiting root zone activity (LeBarron 1945; Islam and Macdonald 2004).

### Species-specific responses

The observed early-establishment responses to the abiotic environment of the five tree species were highly idiosyncratic. The temperature response of germination in the growth chambers revealed a specific pattern for each species (Fig. 3, Table 3), possibly indicating an adaption to different ranges of germination temperatures. These tendencies were confirmed in the field experiment, which further showed contrasting responses to soil moisture and temperature among all studied species. Consequently, explaining and understanding observed patterns in the regeneration limitations of local treeline tree populations requires the consideration of tree species identity (Wardle 1985; Ball et al. 1991; Sullivan and Sveinbjörnsson 2010; Dufour-Tremblay et al. 2012) and a detailed connection to the ecology of each individual species. Hence, in the following we present a tailored, species by species interpretation of the results.

The results of *L.* *decidua* match the ecological features of a typical subalpine, high-elevation tree species with a high tolerance to cold conditions (see Rameau et al. 1993; Brändli 1998). This was reflected in the relatively high success of germination down to 4 °C in the growth chambers and the absence of a temperature response for both early life-stages in the field. Soil moisture, on the other hand, positively affected seedling emergence and survival (Fig. 2A and F), which can be related to the increased water demand and low water use efficiency of the deciduous life-form compared to evergreen conifers (Matyssek 1986). This feature seems to be already inherent to the earliest stages of regeneration, even though first-year seedlings are not deciduous yet.

In contrast, soil moisture had a negative effect on both early life-stages of *P.* *cembra*. For seedling emergence this negative moisture effect was reduced at high temperatures. Although this appears surprising at first, it can be explained by a combination of limiting biotic factors and the life-history strategy of this species. First, *P. cembra* is the highest-occurring tree species in Europe and mostly occurs on steep sloping terrain where moisture limitations are most severe (Brändli 1998). This is not only due to its higher tolerance to the harsh subalpine conditions, but also caused by its low competitive capacities in relation to other high-elevation tree species (Ulber et al. 2004). Second, seed dispersal relies on the nutcracker (*Nucifraga caryocatactes*), a bird hiding seeds specifically in shallow caches in open, wind-exposed (Kajimoto et al. 1998) and early snow-free (Mattes 1994) sites. Both of these aspects suggest that *P. cembra* is well adapted to rather dry regeneration sites, as corroborated by impressively deep tap roots already present in small seedlings (Hättenschwiler and Körner 1995, up to 20 cm in 1-year old plants, i.e. nearly 4-fold the aboveground plant height, personal observation). Such a rooting system might, however, be disadvantageous as soil moisture increases since deeper roots aggravate problems associated with insufficient aeration and cool soil temperatures (Scott et al. 1987). Furthermore, seedlings are highly vulnerable to snow fungi promoted by prolonged snow cover (Senn 1999) and in a previous germination trial (data not shown) we observed high seed mortality due to fungal pathogens. Both findings indicate a general susceptibility of early life-stages to pathogens under high moisture conditions.

The contrasting responses of early establishment in the other two conifers, *P.* *abies* and *P.* *uncinata*, can be directly related to their respective distributional range. In growth chambers and in the field, the germination response of *P. abies* was not or only weakly affected by colder conditions (Figs. 2C and 3B), which is supported by previous studies reporting germination responses temperatures as low as 2 °C (Løken 1959). Seedling survival, however, responded to an interaction of temperature and moisture, with increasing soil moisture compensating a negative effect of high temperatures (Fig. 2H). These findings are in line with the ecological requirements of this boreal-subalpine tree species, tolerating a wide amplitude of environmental conditions except drought stress, which is reflected in its absence from the south side or continental ranges of the European Alps (Rameau et al. 1993; Brändli 1998).

*P.* *uncinata*, on the other hand, is a heliophile subalpine tree species with a southern distribution (Pyrenees, southern European Alps, Rameau *et al.* 1993) and accordingly its germination was strongly limited by lower temperatures (Fig. 3C) and responded positively to higher temperatures under sufficient soil-moisture conditions (Fig. 2D). Seedling survival was not affected by the manipulated microclimatic gradients, which is in line with the high tolerance of *P. uncinata* to drought and a relatively robust seedling stage (Rameau et al. 1993; Batllori and Camarero 2009).

Finally, the only broad-leaved and distributionally ubiquitous species *S.* *aucuparia* displayed the counterintuitive response of both early establishment stages being negatively affected by increasing temperatures. This effect may partly be explained by a limitation of our study design, in which the plots with shading roofs (passive cooling) had the coolest temperatures but also an important change in light conditions. In an additional analysis removing these plots, we showed that the negative temperature effects on the performance of *S. aucuparia* disappeared (**See Supporting Information—Table S2;** importantly, removing these plots did not change the general effects in the conifer species), suggesting that those negative effects were actually an artefact caused by an increased performance under shaded conditions. This is supported by the literature, reporting evidence for shade-tolerant seedlings in *S. aucuparia* (Raspé et al. 2000; Zywiec and Ledwoń 2008). However, in the case of seedling emergence, a significant block effect (Table 2) indicated a true temperature effect, i.e. higher emergence with earlier snowmelt, which means colder temperatures during germination. Furthermore, there is a trend towards higher seedling emergence in the low-temperature plots even when considering only the reduced gradient (C and C + W, **see Supporting Information—Fig. S1**). The negative temperature response for seedling emergence is supported by the germination ability of *S. aucuparia* at temperatures as low as 2 °C (Barclay and Crawford 1984), while negative temperature effects on germination have also been found previously for alpine plant species (Hoyle et al. 2013). *S.* *aucuparia*, which possesses traits of both pioneer and climax species (Zywiec et al. 2013), might benefit from increased germination at low temperatures in two ways: First, low temperatures could act as an additional germination cue to increase germination under conditions suitable for seedling establishment (i.e. shade) and second, it might favour an early germination time to avoid competing with faster growing species.

### Implications for local treeline patterns and dynamics

The regeneration responses found in our study may offer an explanation for observed patterns and dynamics of treeline tree populations, including treeline form and landscape patterns in treeline position and tree population dynamics. For example, the re-invasion of abandoned subalpine pastures by trees was shown to be restricted to colluvial soils alongside forest edges for *L. decidua*, while being concentrated in convex relief forms for *P. cembra* (Didier 2001). According to our results, this may well be due to the respective early-establishment soil-moisture requirements of these species (Fig. 2A, B, F and G). As another example, the positive response of germination to higher temperatures in *P. uncinata* may at least partly explain the increased recruitment of this species observed in the Spanish Pyrenees during relatively warm periods in past centuries and since the 1980s (Camarero et al. 2015). The interaction between temperature and moisture in our experiment suggest that under progressive climate warming, drier conditions might at some point counteract positive warming effects, but so far moisture does not appear to be limiting establishment in this species (Battlori et al 2010). While *P. uncinata* is known to be particularly drought resistant (Rameau et al. 1993), boreal spruce forests have already been shown to suffer increasingly from temperature-induced drought stress (Barber et al. 2000). On the other hand, *P.* *abies* treelines have been observed to advance considerably in the past three decades under concurrent climatic warming (Kullman and Öberg 2009). We found an important interaction between temperature and soil moisture for seedling survival of *P. abies* (Fig. 2H). Hence, depending on local changes in precipitation, growth and recruitment of high-elevation populations of this species could become restricted by a warming climate even though they were, until recently, positively affected by it (Bolli et al. 2007). Note, however, that directly linking germination and seedling behaviour to local treeline features is generally difficult for two reasons: (i) germination and first-year survival are necessary but not sufficient feats to assure tree establishment, and (ii) for the studied species, many treelines that they form are subject to intense anthropogenic influences and are currently not at their climatic limit (Wick and Tinner 1997). Land use can thus be a primary driver of their spatial pattern and recent dynamics, in particular in the European Alps (Didier 2001; Bolli et al. 2007; Vittoz et al. 2008). Similar situations exist for other tree species in many mountains of the world. Therefore, in addition to climatic factors, land-use history needs to be taken into account in observational studies of treeline dynamics.

Our results can also be linked to the important contribution that species-specific requirements of the earliest life-stages exert on the shape and dynamic of a local treeline (Harsch and Bader 2011). For example, if young seedlings require shade or shelter—as did *S. aucuparia* in our study—they will be most successful near existing trees and treeline tree populations will tend to occur in clustered spatial patterns (Smith et al. 2003). Conversely, species requiring increased temperature or light conditions—such as *P. cembra* and *P. uncinata* according to our results (Fig. 2)—may perform better in open microsites and their treeline populations may develop a scattered distribution (Holtmeier 2009). Consequently, abrupt treelines, if not caused by disturbances, are primarily explained by high seedling mortality beyond the forest edge and less so by growing season temperatures, which makes them less responsive to the current climate change. In diffuse treelines in contrast, growth is more and more limited by temperature with increasing elevation or latitude, and accordingly most treeline advances can be expected in this treeline type (Harsch and Bader 2011).

## Conclusions

Recruitment as a population bottleneck plays a crucial role in the discussion about driving forces and future dynamics of treeline ecotones. To our knowledge this is the first study to link the two earliest stages of tree establishment in a multi-species approach experimentally manipulating two potentially limiting microclimatic variables. We show that responses are highly idiosyncratic, but generally consistent over both life-stages within each species, which increases the impact of limiting climate variables in a relatively stable environment. Furthermore, interactions of temperature and moisture highlight the complex interplay of microclimatic factors influencing the regeneration success and confirm the importance of other factors than temperature, such as water availability, for the understanding of treeline dynamics. Our study contributes to the understanding of species-specific requirements and limitations of the vulnerable stages of early establishment, which can be used to explain current treeline patterns and predict future responses in the context of their local climatic conditions.

## Sources of Funding

Our work was funded by the German Research Foundation (DFG; Germany; BA 3843/5-1&2).

## Contributions by the Authors

H.L. designed the experimental details and carried out the field work, data analysis and manuscript preparation, G.Z. contributed to the project design and manuscript preparation, M.Y.B. designed the project and contributed to the article preparation.

## Conflicts of Interest Statement

None declared.

## Supporting Information

The following additional information is available in the online version of this article —

 **Figure S1.** Proportions of seedling emergence and subsequent survival (mean ± SD; *n* = 3) at the end of the first growing season for five treeline tree species grown in a field experiment with manipulations of soil moisture and soil temperature in the French Alps at 2100 m a.s.l.. Treatment abbreviations indicate: C = Control, W = watered control, OTC = passive warming (open top chamber), OTC + W = watered passive warming, Sh = passive cooling (shading roof). Different letters stand for, where present, significant differences. Note that there are no error bars for emergence of *Pinus cembra*, OTC (C), because the number of seedlings was the same in all three replicates.

 **Table S2**. Summary of binomial GLM testing the effect of soil microclimate (soil moisture content, soil growing degree days) for reduced gradients (excluding plots with shading roofs) on the proportions of seedling emergence and subsequent survival at the end of the growing season 2014 of five treeline tree species grown in a field experiment in the french alps at 2100 m.

## References

[plw053-B1] Ball MC Hodges VS Laughlin GP. 1991 Cold-induced photoinhibition limits regeneration of snow gum at a tree-line. Functional Ecology 5:663–668.

[plw053-B2] Barbeito I Dawes MA Rixen C Senn J Bebi P. 2012 Factors driving mortality and growth at treeline: a 30-year experiment of 92 000 conifers. Ecology 93:389–401. 22624320 10.1890/11-0384.1

[plw053-B3] Barber VA Juday GP Finney BP. 2000 Reduced growth of Alaskan white spruce in the twentieth century from temperature-induced drought stress. Nature 405:668–673. 10864320 10.1038/35015049

[plw053-B4] Barclay AM Crawford RMM. 1984 Seedling emergence in the rowan (*Sorbus aucuparia*) from an altitudinal gradient. Journal of Ecology 72:627–636.

[plw053-B5] Barton AM. 1993 Factors controlling plant distributions: drought, competition, and fire in montane pines in Arizona. Ecological Monographs 63:367–397.

[plw053-B6] Baskin CC Baskin JM. 2001 Seeds: ecology, biogeography, and evolution of dormancy and germination, 2nd edn San Diego: Academic Press.

[plw053-B7] Batllori E Camarero JJ. 2009 Seedling recruitment, survival and facilitation in alpine *Pinus uncinata* tree line ecotones. Implications and potential responses to climate warming. Global Ecology and Biogeography 18:460–472.

[plw053-B8] Batllori E Camarero JJ Gutiérrez E. 2010 Current regeneration patterns at the tree line in the Pyrenees indicate similar recruitment processes irrespective of the past disturbance regime. Journal of Biogeography 37:1938–1950.

[plw053-B9] Bolli JC Rigling A Bugmann H. 2007 The influence of changes in climate and land-use on regeneration dynamics of Norway spruce at the treeline in the Swiss Alps. Silva Fennica 41:55–70.

[plw053-B10] Brändli U-B. 1998 Die häufigsten waldbäume der Schweiz - ergebnisse aus dem landesforstinventar 1983-85: verbreitung, standort und haufigkeit von 30 baumarten, 2nd edn Birmensdorf, Switzerland: Eidgenössische Forschungsanstalt tür Wald, Schnee und Landschaft.

[plw053-B11] Camarero JJ García-Ruiz JM Sangüesa-Barreda G Galván JD Alla AQ Sanjuán Y Beguería S Gutiérrez E. 2015 Recent and intense dynamics in a formerly static Pyrenean treeline. Arctic, Antarctic, and Alpine Research 47:773–783.

[plw053-B12] Case BS Duncan RP. 2014 A novel framework for disentangling the scale-dependent influences of abiotic factors on alpine treeline position. Ecography 37:838–851.

[plw053-B13] Castanha C Torn MS Germino MJ Weibel B Kueppers LM. 2012 Conifer seedling recruitment across a gradient from forest to alpine tundra: effects of species, provenance, and site. Plant Ecology and Diversity 6:1–12.

[plw053-B14] Choler P Michalet R Callaway RM. 2001 Facilitation and competition on gradients in alpine plant communities. Ecology 82:3295–3308.

[plw053-B15] Cuevas JG. 2000 Tree recruitment at the *Nothofagus pumilio* alpine timberline in Tierra del Fuego, Chile. Journal of Ecology 88:840–855.

[plw053-B16] Cui M Smith WK. 1991 Photosynthesis, water relations and mortality in *Abies-lasiocarpa* seedlings during natural establishment. Tree Physiology 8:37–46. 14972895 10.1093/treephys/8.1.37

[plw053-B17] Daniels LD Veblen TT. 2004 Spatiotemporal influences of climate on altitudinal treeline in northern Patagonia. Ecology 85:1284–1296.

[plw053-B18] Didier L. 2001 Invasion patterns of European larch and Swiss stone pine in subalpine pastures in the French Alps. Forest Ecology and Management 145:67–77.

[plw053-B19] Donohue K Rubio de Casas R Burghardt L Kovach K Willis CG. 2010 Germination, postgermination adaptation, and species ecological ranges. Annual Review of Ecology, Evolution, and Systematics 41:293–319.

[plw053-B20] Dufour-Tremblay G de Vriendt L Lévesque E Boudreau S. 2012 The importance of ecological constraints on the control of multi-species treeline dynamics in Eastern Nunavik, Québec. American Journal of Botany 99:1638–1646. 22984093 10.3732/ajb.1200279

[plw053-B21] Elliott GP Cowell CM. 2015 Slope aspect mediates fine-scale tree establishment patterns at upper treeline during wet and dry periods of the 20th century. Arctic, Antarctic, and Alpine Research 47:681–692.

[plw053-B22] Ferrar P Cochrane P Slatyer R. 1988 Factors influencing germination and establishment of *Eucalyptus pauciflora* near the alpine tree line. Tree Physiology 4:27–43. 14972833 10.1093/treephys/4.1.27

[plw053-B23] Germino MJ Smith WK. 1999 Sky exposure, crown architecture, and low-temperature photoinhibition in conifer seedlings at alpine treeline. Plant Cell and Environment 22:407–415.

[plw053-B24] Germino MJ Smith WK Resor AC. 2002 Conifer seedling distribution and survival in an alpine-treeline ecotone. Plant Ecology 162:157–168.

[plw053-B25] González de Andrés E Camarero JJ Büntgen U. 2015 Complex climate constraints of upper treeline formation in the Pyrenees. Trees 941–952.

[plw053-B26] Greenwood S Chen J-C Chen C-T Jump AS. 2015 Temperature and sheltering determine patterns of seedling establishment in an advancing subtropical treeline. Journal of Vegetation Science 26:711–721.

[plw053-B27] Grubb PJ. 1977 The maintenance of species-richness in plant communities: the importance of the regeneration niche. Biological Reviews 52:107–145.

[plw053-B28] Harper JL. 1977 Population biology of plants. London, New York: Academic Press.

[plw053-B29] Harsch MA Bader MY. 2011 Treeline form - a potential key to understanding treeline dynamics. Global Ecology and Biogeography 20:582–596.

[plw053-B30] Harsch MA Hulme PE McGlone MS Duncan RP. 2009 Are treelines advancing? A global meta-analysis of treeline response to climate warming. Ecology Letters 12:1040–1049. 19682007 10.1111/j.1461-0248.2009.01355.x

[plw053-B31] Hättenschwiler S Körner C. 1995 Responses to recent climatewarming of *Pinus-sylvestris* and *Pinus-cembra* within their montane transition zone in the Swiss Alps. Journal of Vegetation Science 6:357–368.

[plw053-B32] Holtmeier FK. 2009 Mountain timberlines - ecology, patchiness, and dynamics, 2nd edn Dordrecht: Springer Netherlands.

[plw053-B33] Holtmeier FK Broll G. 2005 Sensitivity and response of northern hemisphere altitudinal and polar treelines to environmental change at landscape and local scales. Global Ecology and Biogeography 14:395–410.

[plw053-B34] Hoyle GL Venn SE Steadman KJ Good RB McAuliffe EJ Williams ER Nicotra AB. 2013 Soil warming increases plant species richness but decreases germination from the alpine soil seed bank. Global Change Biology 19:1549–1561. 23505066 10.1111/gcb.12135

[plw053-B35] Islam MA Macdonald SE. 2004 Ecophysiological adaptations of black spruce (*Picea mariana*) and tamarack (*Larix laricina*) seedlings to flooding. Trees - Structure and Function 18:35–42.

[plw053-B36] Johnson DM McCulloh KA Reinhardt K. 2011 The earliest stages of tree growth: development, physiology and impacts of microclimate In: Meinzer FC Lachenbruch B Dawson TE, eds. Size- and age-related changes in tree structure and function. Dordrecht: Springer Netherlands, 65–87.

[plw053-B37] Kajimoto T Onodera H Ikeda S Daimaru H Seki T. 1998 Seedling establishment of subalpine stone pine (*Pinus pumila*) by nutcracker (*Nucifraga*) seed dispersal on Mt. Yumori northern Japan. Arctic and Alpine Research 30:408–417.

[plw053-B38] Klein JP Moeschberger ML. Modifications by Yan J. 2012 KMsurv: Data sets from Klein and Moeschberger (1997), Survival Analysis. R package version 0.1-5. URL: http://cran.r-project.org/package= KMsurv (Accessed July 18, 2016).

[plw053-B39] Körner C Paulsen J. 2004 A world-wide study of high altitude treeline temperatures. Journal of Biogeography 31:713–732.

[plw053-B40] Kroiss SJ HilleRisLambers J. 2015 Recruitment limitation of long-lived conifers: implications for climate change responses. Ecology 96:1286–1297. 26236842 10.1890/14-0595.1

[plw053-B41] Kullman L. 2007 Tree line population monitoring of *Pinus sylvestris* in the Swedish Scandes, 1973-2005: implications for tree line theory and climate change ecology. Journal of Ecology 95:41–52.

[plw053-B42] Kullman L Öberg L. 2009 Post-Little Ice Age tree line rise and climate warming in the Swedish Scandes: a landscape ecological perspective. Journal of Ecology 97:415–429.

[plw053-B43] LeBarron RK. 1945 Adjustment of black spruce root systems to increasing depth of peat. Ecological Society of America 26:309–311.

[plw053-B44] Lloyd AH Bunn AG. 2007 Responses of the circumpolar boreal forest to 20th century climate variability. Environmental Research Letters 2:045013–0413pp.

[plw053-B45] Løken A. 1959 [Germination experiments in refrigerator chamber]. Meddedels. F. Vestlandets Forstl. Forsøksstasjon 11:1–19.

[plw053-B46] Marion G Henry G Freckman D Johnstone J Jones G Jones M Lévesque E Molau U Molgaard P Parsons A Svoboda J Virginia R. 1997 Open-top designs for manipulating field temperature in high-latitude ecosystems. Global Change Biology 3:20–32.

[plw053-B47] Mattes H. 1994. Coevolutional aspects of stone pines and nutcrackers. In: Schmidt WC, Holtmeier FK, eds. International workshop on subalpine stone pines and their environment: the status of our knowledge. INT-GTR-309. St. Moritz, Switzerland, September 5–11, 1992. UT, USA: Intermountain Research Station, 31–35.

[plw053-B48] Matyssek R. 1986 Carbon, water and nitrogen relations in evergreen and deciduous conifers. Tree Physiology 2:177–187. 14975852 10.1093/treephys/2.1-2-3.177

[plw053-B49] McNair JN Sunkara A Frobish D. 2012 How to analyse seed germination data using statistical time-to-event analysis: non-parametric and semi-parametric methods. Seed Science Research 22:77–95.

[plw053-B50] Moyes AB Castanha C Germino MJ Kueppers LM. 2013 Warming and the dependence of limber pine (*Pinus flexilis*) establishment on summer soil moisture within and above its current elevation range. Oecologia 171:271–282. 22875149 10.1007/s00442-012-2410-0

[plw053-B51] Moyes AB Germino MJ Kueppers LM. 2015 Moisture rivals temperature in limiting photosynthesis by trees establishing beyond their cold-edge range limit under ambient and warmed conditions. New Phytologist 207:1005–1014. 25902893 10.1111/nph.13422

[plw053-B52] Ohse B Jansen F Wilmking M. 2012 Do limiting factors at Alaskan treelines shift with climatic regimes? Environmental Research Letters 7:015505.

[plw053-B53] Ozenda P. 1988 Die vegetation der alpen im europäischen gebirgsraum. Stuttgart; New York: Gustav Fischer Verlag.

[plw053-B54] Qi Z Liu H Wu X Hao Q. 2015 Climate-driven speedup of alpine treeline forest growth in the Tianshan Mountains, Northwestern China. Global Change Biology 21:816–826. 25099555 10.1111/gcb.12703

[plw053-B55] R Core Team. 2015 R: A Language and Environment for Statistical Computing. R version 3.2.1. URL: http://www.r-project.org/ (Accessed July 18, 2016).

[plw053-B56] Rameau J Dumé G Mansion D. 1993 Flore forestiére française: guide écologique illustré. 2. Montagnes. Paris: Institut pour le Développement Forestier.

[plw053-B57] Raspé O Findlay C Jacquemart AL. 2000 Sorbus Aucuparia L. Journal of Ecology 88:910–930.

[plw053-B58] Rolland C Petitcolas V Michalet R. 1998 Changes in radial tree growth for *Picea abies*, *Larix decidua*, *Pinus cembra* and *Pinus uncinata* near the alpine timberline since 1750. Trees 13:40–53.

[plw053-B59] Schupp EW. 1995 Seed-seedling conflicts, habitat choice, and patterns of plant recruitment. American Journal of Botany 82:399–409.

[plw053-B60] Scott P Bentley C Fayle D Hansell R. 1987 Crown forms and shoot elongation of white spruce at the treeline, Churchill, Manitoba, Canada. Arctic and Alpine Research 19:175–186.

[plw053-B61] Senn J. 1999 Tree mortality caused by *Gremmeniella abietina* in a subalpine afforestation in the central Alps and its relationship with duration of snow cover. European Journal of Forest Pathology 29:65–74.

[plw053-B62] Shen W Zhang L Liu X Luo T. 2014 Seed-based treeline seedlings are vulnerable to freezing events in the early growing season under a warmer climate: Evidence from a reciprocal transplant experiment in the Sergyemla Mountains, southeast Tibet. Agricultural and Forest Meteorology 187:83–92.

[plw053-B63] Shiyatov SG Terent’ev MM Fomin VV Zimmermann NE. 2007 Altitudinal and horizontal shifts of the upper boundaries of open and closed forests in the polar Urals. Russian Journal of Ecology 38:223–227.

[plw053-B64] Smith WK Germino MJ Hancock TE Johnson DM. 2003 Another perspective on altitudinal limits of alpine timberlines. Tree Physiology 23:1101–1112. 14522716 10.1093/treephys/23.16.1101

[plw053-B65] Stevens GC Fox JF. 1991 The causes of treeline. Annual Review of Ecology and Systematics 22:177–191.

[plw053-B66] Sullivan PF Sveinbjörnsson B. 2010 Microtopographic control of treeline advance in Noatak National Preserve, Northwest Alaska. Ecosystems 13:275–285.

[plw053-B67] Thébault A Clément JC Ibanez S Roy J Geremia RA Pérez CA Buttler A Estienne Y Lavorel S. 2014 Nitrogen limitation and microbial diversity at the treeline. Oikos 123:729–740.

[plw053-B68] Therneau T. 2015 A Package for Survival Analysis in S. R package version 2.38-1. URL: http://cran.r-project.org/package=survival (Accessed July 18, 2016).

[plw053-B69] Tranquillini W. 1979 Physiological ecology of the alpine timberline. Berlin, Heidelberg: Springer.

[plw053-B70] Trant AJ Hermanutz L. 2014 Advancing towards novel tree lines? A multispecies approach to recent tree line dynamics in subarctic alpine Labrador, northern Canada. Journal of Biogeography 41:1115–1125.

[plw053-B71] Ulber M Gugerli F Bozic G. 2004 EUFORGEN technical guidelines for genetic conservation and use for swiss stone pine (Pinus cembra). Rome: International Plant Genetic Resources Institute.

[plw053-B72] Vittoz P Rulence B Largey T Freléchoux F. 2008 Effects of climate and land-use change on the establishment and growth of cembran pine (P*inus cembra* L.) over the altitudinal treeline ecotone in the central Swiss Alps. Arctic, Antarctic, and Alpine Research 40:225–232.

[plw053-B73] Wardle P. 1985 New Zealand timberlines. 1. Growth and survival of native and introduced tree species in the Craigiebum Range, Canterbury. New Zealand Journal of Botany 23:219–234.

[plw053-B74] Wick L Tinner W. 1997 Vegetation changes and timberline fluctuations in the central Alps as indicators of Holocene climatic oscillations. Arctic and Alpine Research 29:445–458.

[plw053-B75] Zurbriggen N Hättenschwiler S Frei ES Hagedorn F Bebi P. 2013 Performance of germinating tree seedlings below and above treeline in the Swiss Alps. Plant Ecology 214:385–396.

[plw053-B76] Zuur AF Ieno EN Walker NJ Saveliev AA Smith GM. 2009 Mixed effects models and extensions in ecology with R. New York: Springer.

[plw053-B77] Zywiec M Ledwoń M. 2008 Spatial and temporal patterns of rowan (*Sorbus aucuparia* L.) regeneration in West Carpathian subalpine spruce forest. Plant Ecology 194:283–291.

[plw053-B78] Zywiec M Holeksa J Wesolowska M Szewczyk J Zwijacz-Kozica T Kapusta P. 2013 *Sorbus aucuparia* regeneration in a coarse-grained spruce forest - a landscape scale. Journal of Vegetation Science 24:735–743.

